# Specialist palliative and end-of-life care for patients with cancer and SARS-CoV-2 infection: a European perspective

**DOI:** 10.1177/17588359211042224

**Published:** 2021-09-02

**Authors:** Gehan Soosaipillai, Anjui Wu, Gino M Dettorre, Nikolaos Diamantis, John Chester, Charlotte Moss, Juan Aguilar-Company, Mark Bower, Christopher CT Sng, Ramon Salazar, Joan Brunet, Eleanor Jones, Ricard Mesia, Amanda Jackson, Uma Mukherjee, Ailsa Sita-Lumsden, Elia Seguí, Diego Ottaviani, Anna Carbó, Sarah Benafif, Rachel Würstlein, Carme Carmona, Neha Chopra, Claudia Andrea Cruz, Judith Swallow, Nadia Saoudi, Eudald Felip, Myria Galazi, Isabel Garcia-Fructuoso, Alvin J. X. Lee, Thomas Newsom-Davis, Yien Ning Sophia Wong, Anna Sureda, Clara Maluquer, Isabel Ruiz-Camps, Alba Cabirta, Aleix Prat, Angela Loizidou, Alessandra Gennari, Daniela Ferrante, Josep Tabernero, Beth Russell, Mieke Van Hemelrijck, Saoirse Dolly, Nicholas J Hulbert-Williams, David J Pinato

**Affiliations:** Cancer Division, University College London Hospitals, London, UK; Cancer Division, University College London Hospitals, London, UK; UCL Cancer Institute, Fitzrovia, London, UK; Department of Surgery and Cancer, Imperial College London, Hammersmith Hospital, London, UK; Medical Oncology, Barts Health NHS Trust, London, UK; Medical Oncology, School of Medicine, Cardiff University, Cardiff, UK; Medical Oncology, Velindre Cancer Centre, Cardiff, UK; Translational Oncology and Urology Research (TOUR), School of Cancer and Pharmaceutical Sciences, King’s College London, London, UK; Medical Oncology, Vall d’Hebron University Hospital and Institute of Oncology (VHIO), Barcelona, Spain; Infectious Diseases, Vall d’Hebron University Hospital, Barcelona, Spain; Department of Oncology and National Centre for HIV Malignancy, Chelsea & Westminster Hospital, London, UK; Cancer Division, University College London Hospitals, London, UK; Department of Medical Oncology, ICO L’Hospitalet, Oncobell Program (IDIBELL), CIBERONC. Hospitalet de Llobregat, Spain; Department of Medical Oncology, Catalan Institute of Oncology, University Hospital Josep Trueta, Girona, Spain; Medical Oncology, Guy’s and St Thomas’ NHS Foundation Trust (GSTT), London, UK; Department of Medical Oncology, Catalan Institute of Oncology, Badalona, Spain; Clinical Trials, Velindre Cancer Centre, Cardiff, UK; Medical Oncology, Barts Health NHS Trust, London, UK; Medical Oncology, Guy’s and St Thomas’ NHS Foundation Trust (GSTT), London, UK; Department of Medical Oncology, Hospital Clinic, Barcelona, Spain; Cancer Division, University College London Hospitals, London, UK; Department of Medical Oncology, Catalan Institute of Oncology, University Hospital Josep Trueta, Girona, Spain; Cancer Division, University College London Hospitals, London, UK; Department of Gynaecology and Obstetrics, Breast Centre and Gynaecological Cancer Centre and CCC Munich, University Hospital Munich, Munich, Germany; Department of Medical Oncology, Catalan Institute of Oncology, University Hospital Josep Trueta, Girona, Spain; Cancer Division, University College London Hospitals, London, UK; Department of Medical Oncology, Hospital Clinic, Barcelona, Spain; Department of Surgery and Cancer, Imperial College London, Hammersmith Hospital, London, UK; Medical Oncology, Vall d’Hebron University Hospital and Institute of Oncology (VHIO), Barcelona, Spain; Department of Medical Oncology, Catalan Institute of Oncology, Badalona, Spain; Cancer Division, University College London Hospitals, London, UK; Department of Medical Oncology, Catalan Institute of Oncology, University Hospital Josep Trueta, Girona, Spain; Cancer Division, University College London Hospitals, London, UK; Department of Oncology and National Centre for HIV Malignancy, Chelsea & Westminster Hospital, London, UK; Cancer Division, University College London Hospitals, London, UK; Haematology Department, ICO Hospitalet, Hospitalet de Llobregat, IDIBELL, Universitat de Barcelona, Spain; Haematology Department, ICO Hospitalet, Hospitalet de Llobregat, IDIBELL, Universitat de Barcelona, Spain; Infectious Diseases, Vall d’Hebron University Hospital, Barcelona, Spain; Department of Haematology, Vall d’Hebron University Hospital and Institute of Oncology (VHIO), Barcelona, Spain; Department of Medical Oncology, Hospital Clinic, Barcelona, Spain; Translational Genomics and Targeted Therapies in Solid Tumours, IDIBAPS, Barcelona, Spain; Department of Infectious Diseases, Internal Medicine, Institut Jules Bordet, Université Libre de Bruxelles, Brussels, Belgium; Division of Oncology, Department of Translational Medicine, University of Piemonte Orientale and Maggiore della Carità Hospital, Novara, Italy; Department of Translational Medicine, Unit of Cancer Epidemiology, CPO-Piemonte, University of Eastern Piedmont, Novara, Italy; Medical Oncology, Vall d’Hebron University Hospital and Institute of Oncology (VHIO), Barcelona, Spain; Translational Oncology and Urology Research (TOUR), School of Cancer and Pharmaceutical Sciences, King’s College London, London, UK; Translational Oncology and Urology Research (TOUR), School of Cancer and Pharmaceutical Sciences, King’s College London, London, UK; Medical Oncology, Guy’s and St Thomas’ NHS Foundation Trust (GSTT), London, UK; Medical Oncology, Guy’s and St Thomas’ NHS Foundation Trust (GSTT), London, UK; Professor of Behavioural Medicine, Centre for Contextual Behavioural Science, School of Psychology, University of Chester, Chritchley Building, Parkgate Road, Chester, Cheshire, CH1 4BJ, UK; Department of Surgery and Cancer, Clinical Senior Lecturer and Consultant Medical Oncologist, Imperial College London, Hammersmith Hospital, Du Cane Road, London, W12 0HS, UK

**Keywords:** cancer, COVID-19, end-of-life (EOL), end-of life care (EOLC), speciality palliative care team (SPCT)

## Abstract

**Background:**

Specialist palliative care team (SPCT) involvement has been shown to improve symptom control and end-of-life care for patients with cancer, but little is known as to how these have been impacted by the COVID-19 pandemic. Here, we report SPCT involvement during the first wave of the pandemic and compare outcomes for patients with cancer who received and did not receive SPCT input from multiple European cancer centres.

**Methods:**

From the OnCovid repository (*N* = 1318), we analysed cancer patients aged ⩾18 diagnosed with COVID-19 between 26 February and 22 June 2020 who had complete specialist palliative care team data (SPCT+ referred; SPCT− not referred).

**Results:**

Of 555 eligible patients, 317 were male (57.1%), with a median age of 70 years (IQR 20). At COVID-19 diagnosis, 44.7% were on anti-cancer therapy and 53.3% had ⩾1 co-morbidity. Two hundred and six patients received SPCT input for symptom control (80.1%), psychological support (54.4%) and/or advance care planning (51%). SPCT+ patients had more ‘Do not attempt cardio-pulmonary resuscitation’ orders completed prior to (12.6% *versus* 3.7%) and during admission (50% *versus* 22.1%, *p* < 0.001), with more SPCT+ patients deemed suitable for treatment escalation (50% *versus* 22.1%, *p* < 0.001). SPCT involvement was associated with higher discharge rates from hospital for end-of-life care (9.7% *versus* 0%, *p* < 0.001). End-of-life anticipatory prescribing was higher in SPCT+ patients, with opioids (96.3% *versus* 47.1%) and benzodiazepines (82.9% *versus* 41.2%) being used frequently for symptom control.

**Conclusion:**

SPCT referral facilitated symptom control, emergency care and discharge planning, as well as high rates of referral for psychological support than previously reported. Our study highlighted the critical need of SPCTs for patients with cancer during the pandemic and should inform service planning for this population.

## Background

Since the start of the pandemic, coronavirus disease 2019 (COVID-19), the viral infection caused by severe acute respiratory syndrome coronavirus 2 (SARS-CoV-2), has been linked to 740,809 deaths across Europe (as of 12 July 2021),^1^ putting an unprecedented strain on international healthcare services.^2^

Previous studies have shown that mortality from COVID-19 is higher for those of an older age and those with co-morbidities.^3^ Since the beginning of the pandemic, the presence of cancer has been linked to an increased risk of developing severe COVID-19, with a 6.2-fold difference in mortality compared with individuals without cancer (5.6% *versus* 0.9%).^4^ The OnCovid study, the largest registry in Europe describing the natural history and outcomes from SARS-CoV-2 infection in patients with cancer, has shown that mortality from COVID-19 in unselected consecutive patients with cancer can be as high as 30%.^5^ Although provision of chemotherapy, targeted therapy and immunotherapy did not worsen mortality.^6^ Patients with COVID-19 often suffer from debilitating symptoms, such as fever, cough and dyspnoea.^4^ Specialist palliative care team (SPCT) support may be beneficial for patients with advanced malignancies and COVID-19 to control their symptoms as well as provide individualised end-of-life care.^7^ The provision of specialist palliative and end-of-life care for patients can be challenging when services are under-resourced,^7^ independent of the challenges inherent during a pandemic.

Accumulating evidence shows that the early involvement of SPCT for patients with advanced cancer improves quality of life by providing specialist symptom control and support with advance care planning and end-of-life care.^8^ The majority of patients with cancer who acquire SARS-CoV-2 present with debilitating symptoms including fevers, dyspnoea and fatigue, and nearly two-thirds of them rapidly evolve into life threatening disease,^6^ with a high proportion of respiratory failure and end organ damage sustained by the pro-inflammatory response elicited against the virus.^9,10^ Whilst a number of studies, including OnCovid, have extensively documented survival outcomes of patients with COVID-19 and cancer, the trajectory of decline and symptomatic burden that SARS-CoV-2-infected patients with cancer experience from the diagnosis of COVID-19 towards the end-of-life remain to be understood^4,11^ and must be fully characterised to enable effective symptom control.

In addition, whilst patients with cancer and concomitant COVID-19 may benefit from SPCT input to address their symptomatic needs,^12^ questions remain regarding the pandemic’s impact on services and provision of palliative and end-of-life care in this patient subgroup. Whilst studies have been conducted to understand how palliative care services have rapidly responded to those who have been affected by COVID-19,^13–16^ such as providing education and protocols for symptom control and end-of-life care for non-specialist healthcare practitioners, leading psychological support and bereavement care services, and utilising community services, little is known with concern to their specific role in patients with cancer. The pandemic has reinforced the importance of individualised emergency care planning (i.e. treatment escalation planning and cardio-pulmonary resuscitation decisions) by forcing physicians to consider what is important to the patient weighed against the availability of resources.^7,17^ However, the translation of this practice for patients with concomitant COVID-19 and cancer is unknown. As COVID-19 continues to impose an ongoing threat to patients with cancer, it is important to develop direct knowledge of the needs of these patients using an evidence-based approach. Here, we aim to describe the demographics of patients with cancer hospitalised with COVID-19, describe the patterns of referral to SPCTs, and compare emergency care planning and care in the last days of life among patients referred to and not referred to SPCTs. To address these aims, we evaluated the natural histories and outcomes of over 500 patients with cancer recruited to the OnCovid study.

## Methods

### Study population, setting and data collection

**Figure 1.**
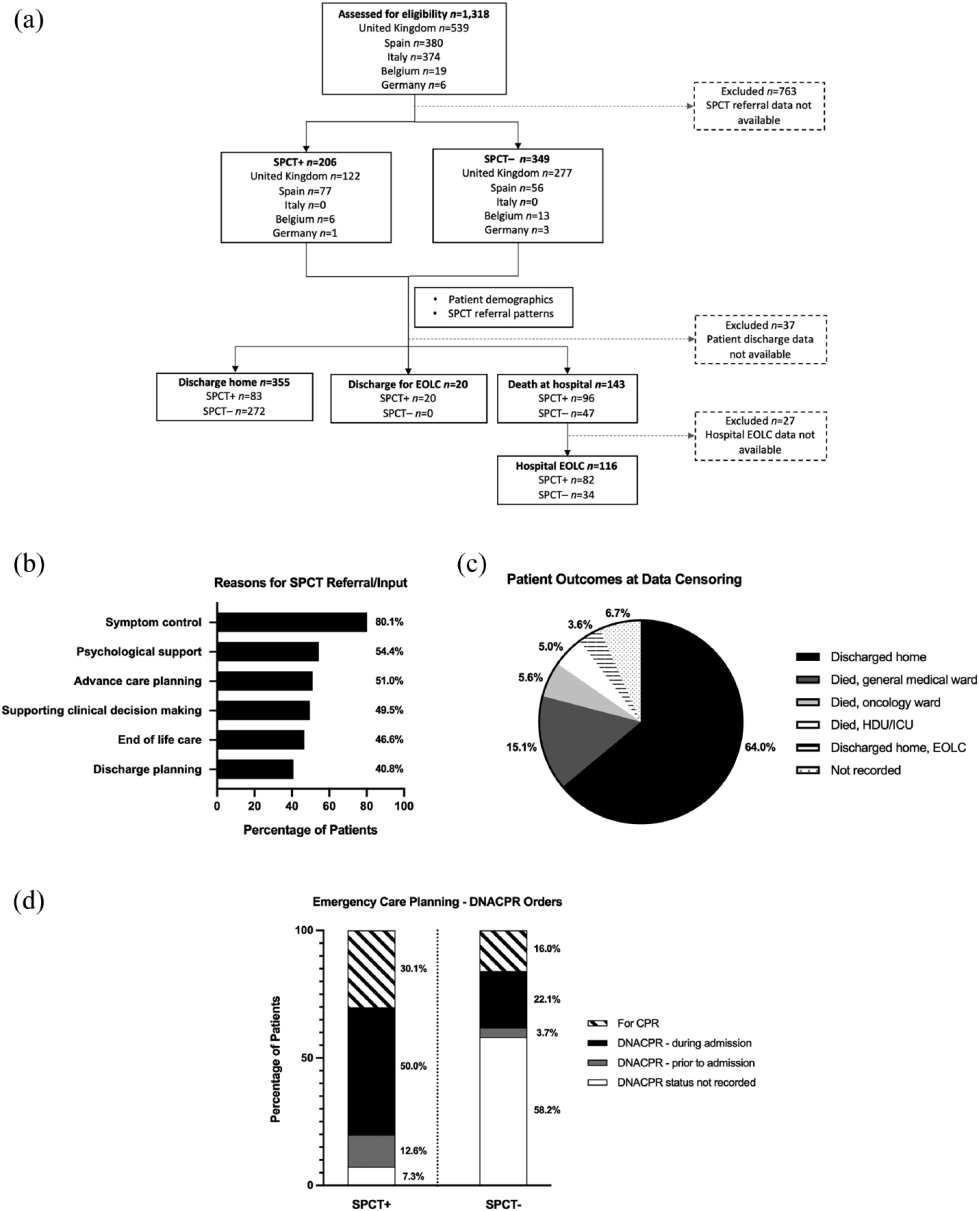
Patient disposition, referral, outcomes and emergency care planning. (a) Study design and patient assortment. (b) Causes for specialist palliative care team (SPCT) involvement (*n* = 206). (c) Outcomes for all eligible patients (*n* = 555). (d) Emergency care planning for patients in the SPCT+ (*n* = 206) and SPCT− (*n* = 349) cohorts. CPR, cardio-pulmonary resuscitation; DNACPR, Do not attempt cardio-pulmonary resuscitation; EOLC, end-of-life care; HDU, high-dependency unit; ICU, intensive care unit; SPCT, specialist palliative care team; SPCT+, SPCT referred; SPCT-, SPCT not referred.

This study focuses on a subset of patients accrued to the main OnCovid registry for whom data regarding SPCT referral was available for analysis. Methodology and clinical outcomes of the first 890 patients included in the main OnCovid study have been previously reported.^6^ Briefly, main eligibility criteria for OnCovid included being ⩾18 years of age, having a confirmed diagnosis of SARS-CoV-2 infection by reverse-transcriptase polymerase chain reaction (RT-PCR) of a nasopharyngeal swab, and history of solid or haematologic malignancy, either active (those receiving anti-cancer treatment) or in remission at the time of COVID-19 diagnosis. Patients with a history of non-invasive/premalignant lesions or with low malignant potential (i.e. basal cell carcinoma of the skin, non-invasive carcinoma *in situ* of the cervix, ductal carcinoma *in situ*) were excluded. For haematologic malignancies, only patients carrying an oncological diagnosis of defined malignant behaviour (lymphoma, leukaemia, multiple myeloma) were included. For the purpose of the current analysis, participating investigators performed an *ad hoc* review of medical records of hospitalised patients for COVID-19 to assess whether or not referral to SPCT was made during hospitalisation. From 26 February 2020 to 22 June 2020, 1318 patients were consecutively accrued to OnCovid across 24 European academic centres. Of these 1318 patients, 555 patients (42%) who had been hospitalised for COVID-19 from 13 European academic centres had complete SPCT referral records and were included in this study [Figure 1(a); Supplemental material Table 1 online]. All patients were observed from the time of COVID-19 diagnosis, defined by SARS-CoV-2 PCR positivity until date of death or, in COVID-19 survivors, date of discharge from hospital or last outpatient follow-up post-discharge.

Within the UK, OnCovid was granted central ethical approval by the Health Research Authority (20/HRA/1608). Outside of the UK, this study was granted ethical approval by the corresponding ethics review boards at each participating site (Supplemental Table 2). Competent authorities waived prospective informed consent due to the retrospective nature of data collection and the use of anonymised data. In order to maintain confidentiality standards, each patient enrolled into the study was assigned a unique pseudonymisation code through assignment of an identification number. Clinical data including patients’ demographics, laboratory tests and radiologic results were reviewed retrospectively by clinicians and collated into a case report form designed using the Research Electronic Data Capture (REDCap, Vanderbilt University) tool hosted by the Medical Statistics Unit in Novara, Italy,^18,19^ which coordinated database access and curation.

Alongside data concerning features of COVID-19, including co-morbidities and requirement for and length of hospitalisation,^6^ we collected timing, reason(s) for referral to the SPCT, patient outcome (discharge or place of death in the hospital setting), symptomatology and use of anticipatory medications (classified as: opioids, benzodiazepines, antipsychotics, antiemetics, antimuscarinics and antipyretics) in the final 72 h of life. All medical records of cases recruited to this study were reviewed by physicians involved in delivering patients’ care, with the final follow-up date for all patients being 22 June 2020.

### Study definitions

The diagnosis of COVID-19 and description of the clinical syndromes associated with the disease, including acute respiratory distress syndrome (ARDS), followed criteria published by the World Health Organization.^20^ All patients recruited to this study were confirmed positive for SARS-CoV-2 infection following RT-PCR testing of nasopharyngeal swab samples using validated methodology. Nosocomial SARS-CoV-2 contraction was defined in patients who developed symptoms and tested positive for COVID-19 whilst admitted to the hospital for other reasons. Recognising the significant heterogeneity in the referral pathways to palliative care across centres and countries, we elected to present patients who were referred to SPCT prior to COVID-19 and those who were referred at the point of COVID-19 diagnosis in a joint category (SPCT+).

### Statistical analysis

Continuous data following non-parametric distribution are presented as median with interquartile range (IQR). Categorical data are described as percentages. To determine statistical significance of results, the Mann–Whitney *U* test was utilised for continuous data following non-parametric distribution and Fisher’s exact test or the chi-squared test employed for analysis of categorical variables.

### Role of the funding source

Wellcome Trust Strategic Fund (PS3416, 2018) and Associazione Italiana per la Ricerca sul Cancro (14230, 2019) provided grant support. Cancer Research UK Imperial Centre and the Imperial NIHR BRC have provided infrastructural support.

## Results

### Patient demographics

Of the 1318 patients within the OnCovid database at data censoring (22 June 2020), 555 patients had SPCT date collected and were eligible for inclusion in this study [Figure 1(a)]. Patient data was submitted by 13 centres from the United Kingdom *(n* = 399, 71.8%), Spain *(n* = 133, 23.9%), Belgium *(n* = 19, 3.4%) and Germany *(n* = 4, 0.7%; Supplemental Table 1). The median follow-up time was 28 days (IQR 47). Most patients were male *(n* = 317, 57.1%) with a median age of 70 years (IQR 20), carried a diagnosis of active malignancy *(n* = 369, 66.5%) and had localised disease *(n* = 229, 41.6%; Table 1). The commonest primary tumour sites were genitourinary *(n* = 132, 23.8%), breast *(n* = 83, 15%) and lung *(n* = 67, 12.1%). The majority of patients had at least one co-morbidity *(n* = 442, 79.6%), most commonly hypertension *(n* = 273, 49.2%) and diabetes *(n* = 131, 23.6%). At COVID-19 diagnosis, 248 (44.7%) patients were on systemic anti-cancer therapy, of whom 57 (10.3%) received therapy with palliative intent; 285 (51.4%) patients were not on active treatment.

**Table 1.** Demographic data of patients with SARS-CoV-2 infection and SPCT referral data.

	SPCT+	SPCT−	Total
	*n* = 206	*n* = 349	*N* = 555
Age, years, median (IQR)	71 (18.75)	68 (20)	70 (20)
Age			
<65 years, *n* (%)	65 (31.6)	135 (38.7)	355 (64.0)
⩾65 years, *n* (%)	141 (68.4)	214 (61.3)	200 (36.0)
Sex, *n* (%)			
Male	99 (48.1)	218 (62.5)	317 (57.1)
Female	106 (51.5)	129 (37)	235 (42.3)
Information unavailable	1 (0.5)	2 (0.6)	3 (0.5)
Smoking history, *n* (%)			
Never smoker	93 (45.1)	157 (45)	250 (45)
Current/former smoker	89 (43.2)	138 (39.5)	259 (46.7)
Unknown	22 (10.7)	49 (14)	46 (8.3)
Cancer type, *n* (%)			
Head and neck	6 (2.9)	11 (3.2)	17 (3.1)
Lung and thoracic	11 (3.2)	31 (8.9)	42 (7.6)
Gastroesophageal	13 (6.3)	10 (2.9)	23 (4.1)
Hepatobiliary	8 (3.9)	10 (2.9)	18 (3)
Duodenal and lower GI tract	25 (12.1)	38 (10.9)	63 (11.4)
Breast	34 (16.5)	49 (14.1)	83 (15.0)
Gynaecological	16 (4.6)	23 (11.2)	39 (7.0)
Genitourinary	38 (18.4)	94 (26.9)	132 (23.8)
Skin	8 (3.9)	18 (5.2)	26 (4.7)
Lymphoma	3 (1.5)	24 (6.9)	27 (4.9)
Other	9 (4.4)	55 (15.8)	64 (11.5)
Tumour stage, *n* (%)			
Localised	52 (25.2)	177 (50.7)	229 (41.6)
Locoregional	29 (14.1)	59 (16.9)	88 (15.8)
Metastatic	120 (58.3)	79 (22.6)	199 (35.9)
Number of metastatic sites			
0	72 (35)	236 (67.6)	308 (55.5)
1	3 (1.5)	4 (1.1)	7 (1.26)
2	57 (27.7)	41 (11.7)	98 (17.66)
⩾3	65 (31.6)	38 (10.9)	103 (18.56)
Unknown	9 (4.4)	30 (8.6)	39 (7.03)
Tumour status at COVID-19 diagnosis, *n* (%)			
Active malignancy	161 (78.2)	208 (59.6)	369 (66.49)
Remission	42 (20.4)	124 (35.5)	166 (29.91)
Unknown	3 (1.5)	17 (4.9)	20 (3.6)
Ongoing anticancer therapy at COVID-19 diagnosis, *n* (%)			
Yes	102 (49.5)	146 (41.8)	248 (44.68)
No	101 (49)	184 (52.7)	285 (51.35)
Unknown	3 (1.5)	19 (5.4)	22 (3.96)
Prior radical therapies, *n* (%)			
Surgery	102 (49.5)	150 (42.9)	252 (45.41)
Adjuvant/neoadjuvant chemotherapy	82 (39.8)	89 (25.5)	171 (30.81)
Prior curative systemic therapy, *n* (%)	7 (3.4)	38 (10.9)	45 (8.11)
Prior radiotherapy, *n* (%)	68 (33)	89 (25.5)	157 (28.29)
Prior palliative systemic therapy, *n* (%)			
Chemotherapy	17 (8.3)	9 (2.6)	26 (4.68)
Immunotherapy	16 (7.8)	4 (1.1)	20 (3.6)
Endocrine therapy	15 (7.3)	7 (2)	22 (3.96)
Targeted therapy	7 (3.4)	7 (2)	14 (2.52)
Ongoing palliative systemic anticancer therapy, *n* (%)			
Yes	77 (37.4)	40 (11.5)	117 (21.08)
No	113 (54.9)	262 (75.1)	375 (67.57)
Unknown	16 (7.8)	47 (13.5)	63 (11.35)
Comorbidities, *n* (%)			
Hypertension	106 (51.5)	167 (47.9)	273 (49.19)
Diabetes	43 (20.9)	88 (25.2)	131 (23.6)
Cardiovascular disease	48 (23.3)	49 (14)	97 (17.48)
Chronic pulmonary disease	34 (16.5)	56 (16)	90 (16.22)
Chronic kidney disease	28 (13.6)	52 (14.9)	80 (14.41)
Cerebrovascular disease	15 (7.3)	34 (9.7)	49 (8.83)
Dementia	16 (7.8)	25 (7.2)	41 (7.39)
Peripheral vascular disease	6 (2.9)	8 (2.3)	14 (2.52)
Liver impairment	2 (1)	12 (3.4)	14 (2.52)
Immunosuppression	9 (4.4)	27 (7.7)	36 (6.49)
Steroid therapy in progress	8 (3.9)	15 (4.3)	23 (4.14)
Other	42 (20.4)	85 (24.4)	127 (22.88)
Number of comorbidities, *n* (%)			
0	37 (20)	76 (21.8)	113 (20.36)
1	55 (26.7)	91 (26.1)	146 (26.31)
2	56 (27.2)	65 (18.6)	121 (21.8)
⩾3	58 (28.2)	117 (33.5)	175 (31.53)
COVID-19 symptoms at diagnosis, *n* (%)			
Fever	125 (60.7)	205 (58.7)	330 (59.46)
Cough	109 (52.9)	186 (53.3)	295 (53.15)
Dyspnoea	30 (14.6)	41 (11.7)	71 (12.79)
Fatigue	43 (20.9)	80 (22.9)	123 (22.16)
Myalgia	19 (9.2)	45 (12.9)	64 (11.53)
Diarrhoea	30 (14.6)	41 (11.7)	71 (12.79)
Coryzal symptoms	21 (10.2)	28 (8.0)	49 (8.83)
Nausea or vomiting	23 (11.2)	27 (7.7)	50 (9.01)
Sore throat	7 (3.4)	3 (1)	10 (1.8)
Headache	7 (3.4)	15 (4.3)	22 (3.96)
Dysgeusia	5 (2.4)	8 (2.3)	13 (2.34)
Anosmia	6 (2.9)	8 (2.3)	14 (2.52)
Other (i.e. confusion, delirium, etc.)	33 (16)	100 (28.7)	133 (23.96)
Number of symptoms at diagnosis, *n* (%)			
0	10 (4.9)	31 (8.9)	41 (7.39)
1	46 (22.3)	70 (20.1)	116 (20.9)
2	60 (29.1)	90 (25.8)	150 (27.03)
⩾3	90 (43.7)	158 (45.3)	248 (44.68)
Hospitalisation rate, *n* (%)			
Community-acquired (self-isolation recommended)	2 (1)	58 (16.6)	60 (10.81)
Community-acquired (admission required)	134 (65)	209 (59.9)	343 (61.8)
Hospital-acquired	69 (33.5)	76 (21.8)	145 (26.13)
Admission to intensive or sub-intensive care unit, *n* (%)	16 (7.8)	45 (12.9)	61 (10.99)
COVID-19-specific drug treatments, *n* (%)			
Antibiotics	93 (45.1)	173 (49.6)	266 (47.93)
Hydroxychloroquine or chloroquine	58 (28.2)	57 (16.3)	115 (20.72)
Systemic corticosteroids	14 (6.8)	20 (5.7)	34 (6.13)
Lopinavir/ritonavir	25 (12.1)	17 (4.9)	42 (7.57)
Remdesivir	5 (2.4)	0 (0)	5 (0.9)
Tocilizumab	8 (3.9)	13 (3.7)	21 (3.78)
Others	9 (4.4)	14 (4.0)	23 (4.14)
COVID-19-specific oxygen interventions, *n* (%)			
Oxygen therapy	132 (64.1)	167 (47.9)	299 (53.87)
Mechanical ventilation	12 (5.8)	33 (9.5)	45 (8.11)
High-flow oxygen therapy	62 (30.1)	82 (23.5)	144 (25.95)
COVID-19 complications, *n* (%)			
Acute cardiac injury	6 (2.9)	13 (3.7)	19 (3.42)
Acute kidney injury	21 (10.2)	28 (8.0)	49 (8.83)
Acute liver injury	2 (1)	5 (1.4)	7 (1.26)
Acute respiratory failure	83 (40.3)	74 (21.2)	157 (28.29)
ARDS	27 (13.1)	35 (10.0)	62 (11.17)
Disseminated intravascular coagulation	2 (1)	2 (1)	4 (0.72)
Secondary infection	21 (10.2)	35 (10.0)	56 (10.09)
Others	6 (2.9)	7 (2)	13 (2.34)
Number of complications, *n* (%)			
0	90 (43.7)	234 (67)	324 (58.38)
1	78 (37.9)	73 (20.9)	151 (27.21)
2	31 (15)	20 (5.7)	51 (9.19)
⩾3	7 (3.4)	22 (6.3)	29 (5.23)

ARDS, acute respiratory distress syndrome; COVID-19: coronavirus disease 2019; GI, gastrointestinal; IQR, interquartile range; SARS-CoV-2, severe acute respiratory syndrome coronavirus 2; SPCT, specialist palliative care team; SPCT+, SPCT referred; SPCT-, SPCT not referred.

In the 555 eligible patients (Table 1), the most common presenting symptoms were fever *(n* = 330, 59.5%) and cough *(n* = 295, 53.2%). Of the 488 (87.9%) patients admitted to hospitals, ward-based care was deemed appropriate in 133 (24%) patients, whereas escalation to intensive or high-dependency care was deemed necessary in 62 (11.2%) patients. Hospitalisation lasted for a median duration of 10 days (IQR 10.5), whereas median stay in intensive or high-dependency care was 7 days (IQR 12.8). Supplemental oxygen therapy was required for 299 (53.8%) patients, including high-flow delivery for 144 (25.9%) patients. Mechanical ventilation was initiated on 45 patients (8.1%), including non-invasive ventilation *(n* = 33, 5.9%) and endotracheal intubation *(n* = 18, 3.2%). In total, 314 (56.6%) patients received at least one form of treatment for COVID-19, most frequently broad-spectrum antibiotics *(n* = 266, 47.9%), followed by hydroxychloroquine or chloroquine *(n* = 115, 20.7%) and lopinavir/ritonavir *(n* = 42, 7.6%). In total, 234 (42.2%) patients developed complicated COVID-19 disease, defined as the development of acute respiratory failure (ARDS), acute kidney injury, secondary infection, sepsis, septic shock, acute cardiac injury, acute liver injury, or other conditions, including disseminated intravascular coagulation.

### Patterns of referral to SPCTs

Of all 555 eligible patients, 206 patients (37%) were referred to their respective SPCT during the observation time (SPCT+), whereas 349 patients (63%) were not (SPCT−; Figure 1(a)). As described in Table 1, the proportion of patients aged ⩾65 years (SPCT+ *n* = 141, 68.4%; SPCT− *n* = 214, 61.3%; *p* = 0.091) and those with higher co-morbid burden (i.e. ⩾2 co-morbidities) were similar across groups (SPCT+ *n* = 114, 55.4%; SPCT− *n* = 182, 52.1%; *p* = 0.46). Compared with the SPCT− cohort, SPCT+ patients were more likely to have metastatic disease at COVID-19 diagnosis (SPCT+ *n* = 120, 58.3%; SPCT− *n* = 79, 22.6%; *p* < 0.001) and more likely to have developed a greater number of COVID-19 complications during observation (SPCT+ *n* = 38, 18.4%; SPCT− *n* = 42, 12%; *p* = 0.037 between 0 and 1 *versus* ⩾2 COVID-related complications). A significantly larger proportion of SPCT+ patients were undergoing anticancer therapy (SPCT+ *n* = 102, 49.5%; SPCT− *n* = 146, 41.8%; *p* = 0.008) and systemic anticancer therapy with palliative intent (SPCT+ *n* = 77, 37.4%; SPCT− *n* = 40, 11.5%; *p* < 0.001) at COVID-19 diagnosis. Of the 206 SPCT+ patients, the majority had not previously received palliative care and were newly referred to the hospital SPCT *(n* = 147, 71.4%). A smaller proportion of patients had previously received palliative care and were known to both hospital and community teams *(n* = 39, 18.9%) or to community teams only *(n* = 17, 8.3%). Figure 1(b) highlights the most common reasons for SPCT referral, including symptom control *(n* = 165, 80.1%), psychological support *(n* = 112, 54.4%) and/or advance care planning (*n* = 105, 51.0%).

### Outcomes from COVID-19 and emergency care planning

Figure 1(c) depicts the outcomes of SPCT+ patients at data censoring. Of the 555 patients, 202 (36.4%) were deceased at data censoring. The median overall survival from COVID-19 diagnosis to last follow-up was 47 days (IQR 34.5). The unadjusted mortality rate of the SPCT+ group was more than double that of the SPCT− group (SPCT+ *n* = 117, 56.8%; SPCT− *n* = 85, 24.4%; *p* = 0.008). In this study, there were 145 patients with nosocomial SARS-CoV-2 infection (SPCT+ *n* = 69, 33.5%; SPCT− *n* = 76, 21.8%) and 343 patients with community-acquired SARS-CoV-2 (SPCT+ *n* = 134, 65%; SPCT− *n* = 209, 59.9%). Patient outcome from COVID-19 infection (defined as recovery, in hospital mortality or discharge from hospital) was recorded in 518 patients (SPCT+ *n* = 199, 96.6%; SPCT− *n* = 319, 91.4%). In total, 355 patients were discharged home following recovery from COVID-19 (SPCT+ *n* = 83, 40.3%; SPCT− *n* = 272, 59%). Twenty (9.7%) SPCT+ patients were discharged home for end-of-life care, whereas 115 patients died on oncology (SPCT+ *n* = 23, 11.2%; SPCT− *n* = 8, 2.3%) or general medical wards (SPCT+ *n* = 60, 29.1%; SPCT− *n* = 24, 6.9%). Twenty-eight patients died in high-dependency or intensive care units (SPCT+ *n* = 13, 6.3%; SPCT− *n* = 15, 4.3%). The median time from COVID-19 diagnosis to discharge was 9 days (IQR 11), whereas the median time from COVID-19 diagnosis to death amongst in-hospital decedents was 8 days (IQR 9). Emergency care plans, defined as written documentation of an escalation plan or a do not attempt cardio-pulmonary resuscitation (DNACPR) order, were completed for 219 (39.5%) patients. SPCT+ patients had more DNACPR orders completed prior to admission (SPCT+ *n* = 26, 12.6%; SPCT− *n* = 13, 3.7%) and during admission (SPCT+ *n* = 103, 50%; SPCT− *n* = 77, 22.1%; *p* < 0.001; Figure 1(d)). At data censoring, of the 90 SPCT− patients with a DNACPR order, 51 (56.7%) had died. The median number of days from completion of a DNACPR order to death was 3 days (IQR 7.5). Of the 129 SPCT+ patients with a DNACPR order, 99 (76.7%) had died. The median number of days from completion of a DNACPR order to death was 11 days (IQR 19 days).

### Care in the final days of life

**Figure 2.**
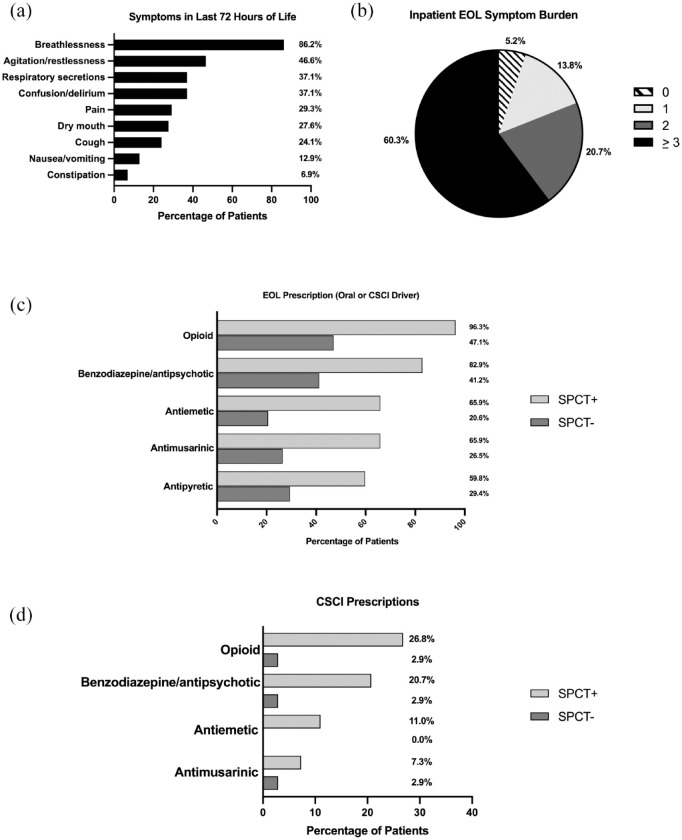
Patient end-of-life symptoms and prescriptions. (a) Symptoms in last 72 h of life for all eligible patients (*n* = 116). (b) End-of-life (EOL) symptom burdens (*n* = 116). (c) EOL prescriptions for SPCT+ (*n* = 82) and SPCT− (*n* = 34) cohorts. (d) CSCI prescriptions for SPCT+ (*n* = 82) and SPCT− (*n* = 34) cohorts. CSCI, continuous subcutaneous infusion; EOL, end-of-life; SPCT, specialist palliative care team; SPCT+, SPCT referred; SPCT-, SPCT not referred.

For all 143 inpatients who were in-hospital decedents, complete data on end-of-life care was available for 116 (SPCT+ *n* = 82, 39.8%; SPCT− *n* = 34, 9.7%). The distribution of symptoms in the last 72 h of life is illustrated in Figure 2(a), with breathlessness (*n* = 100, 86.2%), agitation/restlessness (*n* = 54, 46.6%), confusion/delirium (*n* = 43, 37.1%) and respiratory secretions (*n* = 43, 37.1%) comprising the most common terminal symptoms. The median number of terminal symptoms was 3 (IQR 2), with 70 (60.3%) patients experiencing ⩾3 symptoms in the last days of life (Figure 2(b)).

Given the high burden of end-of-life symptoms, we evaluated patterns of prescription of anticipatory medications. For in-hospital decedents, opioids were most commonly prescribed for pain and breathlessness (SPCT+ *n* = 79, 96.3%; SPCT− *n* = 16, 47.1%), followed by benzodiazepines or antipsychotics for agitation (SPCT+ *n* = 68, 82.9%; SPCT− *n* = 14, 41.2%). Ninety patients were simultaneously prescribed more than one class of symptomatic medication (SPCT+ *n* = 77, 93.9%; SPCT− *n* = 13, 38.2%; median number of classes: SPCT+ 3; SPCT− 0). The vast majority of patients prescribed anticipatory medications were in the SPCT+ cohort [Figure 2(c)]. Of in-hospital decedents with complete end-of-life care data (*n* = 116), continuous subcutaneous infusions (CSCIs) were prescribed for 25 patients (SPCT+ *n* = 24, 29.3%; SPCT− *n* = 1, 2.9%). Opioids constituted the most common class of symptomatic therapy delivered *via* CSCI (SPCT+ *n* = 22, 26.8%; SPCT− *n* = 1, 2.9%), followed by benzodiazepines or antipsychotics (SPCT+ *n* = 17, 20.7%; SPCT− *n* = 1, 2.9%). Figure 2(d) illustrates the distribution of CSCI therapies across SPCT groups.

## Discussion

Whilst increasing research efforts have been dedicated to understanding the impact of COVID-19 in the natural history of patients with cancer,^6^ this is the first observational study investigating specialist palliative care outcomes in this patient population, where guidance on clinical management rests on expert opinions rather than direct evidence.^12^ This is particularly important when considering the potentially increased reliance on hospital-based services in providing psychosocial and supportive care given the closure and limited availability of third-sector face-to-face services through the pandemic.^21^ In recent years, palliative medicine has progressively shifted from a specialty providing care to patients with advanced cancers who do not qualify for active anti-cancer therapy,^22^ or those who are dying,^23^ to a supportive-care service devoted to optimising quality of life alongside active anti-cancer treatment.^24^ However, the relative contribution of palliative care in the context of a highly lethal and often rapidly fatal diagnosis such as COVID-19 has remained relatively unaddressed in patients with cancer.^25^

In this purposely designed sub-study, including 42% of the patients recruited to the OnCovid repository, provision of palliative care by specialised teams was sought in 37% of the accrued patients. Throughout the observation period, patients with active malignancy, metastatic disease, higher tumour burden and higher proportion of COVID-19-related complications were more likely to have received SPCT input, which, in over 70% of the cases, was provided for the first time during inpatient admission. Interestingly, half of the SPCT+ patients were on active anti-cancer therapy at COVID-19 diagnosis. This suggests that a high proportion of patients possessed a good performance status prior to SARS-CoV-2 infection and highlights the impact of COVID-19 as a dominant driver of the acute clinical and symptomatic deterioration leading to instigate palliative care support. Based on our data, symptom control (i.e. breathlessness) was in fact the predominant reason for SPCT referral in over 80% of our patients, most of whom suffered from a multitude of symptoms as a likely consequence of higher tumour burden and higher complication rates from COVID-19.

The second leading cause instigating SPCT review was psychological support. This is a particularly interesting finding given that previous studies demonstrate SPCT referral for emotional and psychological support to be much less frequently cited reasons for referral: previous literature from Japan^26^ and Australia^27^ identified much lower referral rates for emotional issues (22% and <40% respectively) than those found in our analysis (54.4%). It is possible that the increase observed during the pandemic is related to the fact that many of these patients with cancer are being cared for outside of oncology and palliative wards and thus healthcare staff in these different settings may feel less prepared to deal directly with the emotional and psychological issues at end-of-life compared with the specialist oncology workforce. Furthermore, anxiety has been shown to be prevalent amongst hospitalised patients due to isolation from families and fear of deterioration.^28^

It is important also to be cognisant that a paramount component of the ethos of SPCTs is to provide psychological support not only to the patient but also to their families and loved ones.^22^ In the case of cancer, patients’ families may be expecting this support towards end-of-life. Where COVID-19 infection has prompted an unexpectedly rapid health decline, that usual level of psychosocial and emotional support for family members may be difficult – if not impossible – to access. SPCTs will be more aware of this and, perhaps, more able to provide a heightened level of support for these patients’ families.

An important aim of our research was to describe emergency care planning in patients with cancer in the context of a COVID-19 diagnosis, a theme of high clinical interest given the unprecedented strain on healthcare systems imposed by a rapidly diffusing infection with heightened strain on intensive care capacity at the peak of the SARS-CoV-2 pandemic, posing difficult ethical issues of healthcare rationing.^17^ Clear documentation of a designated treatment escalation plan is of utmost importance in patients with cancer as it prevents distressing or unnecessary investigations that are inappropriate in patients with limited life expectancy, whilst on the other hand recognises circumstances where aggressive medical treatment and end organ support are warranted where chances of recovery are reasonable.^29,30^ SPCTs have been shown to help facilitate and lead this decision-making process, especially when patients are being primarily cared for by generalist staff.^31^

Careful review of patients’ records revealed that >90% of SPCT+ patients had documented evidence of an escalation plan compared with approximately 40% of SPCT− patients. Whilst it may be argued that the higher frailty of the SPCT+ subgroup might have favoured clinicians’ increased engagement in DNACPR discussions with SPCTs, our data surprisingly demonstrate that almost one-third of SPCT+ patients were deemed appropriate for CPR during admission. Whilst it should be remembered that our study is a retrospective account of routine clinical practice during the COVID-19 pandemic, we believe this to be a clinically important finding as it suggests that SPCT input in the context of the multi-disciplinary team is essential not only to prevent futile interventions in clinical care but also to support clinical decision making and address the needs of patients whose clinical deterioration is deemed reversible.

In cases where SPCT support was sought, we noted a significantly longer interval between DNACPR order completion and death compared with patients with no documented SPCT input, highlighting that SPCT involvement may facilitate earlier end-of-life care discussions and planning, avoiding treatment escalation decisions in the final days of life, a time in which involvement of patients and relatives becomes increasingly difficult and potentially distressing.^32^

A further aim of our study was to describe patterns of deterioration and symptomatic burden in patients who succumbed to COVID-19. Interestingly, our study shows that the vast majority of in-hospital deaths occurred in clinical areas not specifically dedicated to the care of oncology patients (i.e. emergency areas, medical wards, intensive care, COVID-19 isolation wards). This is an important finding giving that preferred place of death for patients with cancer is usually either a specialist palliative care (hospice) setting^33^ or at home,^34^ and that those patients with cancer who die in hospital or intensive care units typically experience greater emotional distress and poorer quality of end-of-life.^35^ Death in a hospital setting is likely appropriate where symptom burden is higher, and the increase of deaths in the hospital setting during the first wave^36^ is known to have negatively impacted caregiver bereavement outcomes when compared with death at home.^37^ This is especially relevant in the case of COVID-19 related deaths where access to SPCT for families may be reduced more than usual.

In addition, symptom burden in the last days of life was prevalent, with breathlessness and agitation being the most prevalent symptoms in the final hours of life, reflecting the symptoms experienced by a non-selected population of patients dying with COVID-19.^38^ The majority of in-hospital decedents displayed multiple symptoms, highlighting the complex symptomatic needs of this patient population. Consequently, most patients required more than one therapeutic class of symptomatic agent, including opioids to reduce breathlessness and pain, and benzodiazepines or neuroleptics to address terminal restlessness. Generalist medical staff may lack confidence in the prescription of anticipatory end-of-life medications, and the support of SPCTs can ensure adequate higher dose prescriptions to meet patients’ symptomatic needs.^39^ Taken together, these findings further reinforce that the involvement of SPCT is crucial in patients with cancer who have a high symptomatic burden, as this allows (i) adequate recognition of deteriorating patients, (ii) judicious and effective anticipatory prescribing, and (iii) better management of psychosocial concerns, leading to improved quality of life and affective state.^24,40,41^ Our study is consistent with previous knowledge in this field as it highlights more prevalent use of pharmacologic symptomatic care in patients with access to SPCT input.^42^ This is particularly true when we consider prescription of CSCI, a safe and effective drug administration route that can optimise symptom control in patients who cannot tolerate oral medications. Perhaps unsurprisingly, prescription of CSCI was significantly higher in the SPCT+ cohort in our study.

OnCovid and other studies have shown that the mortality from SARS-CoV-2 can be as high as 30% in patients with cancer.^5,43^ Meeting preferred place of end-of-life care can be challenging in a pandemic due to risk of transmission and an unpredictable course of patient deterioration. Here, we show that planning of domiciliary end-of-life care was possible in 10% of patients, all of whom had received input from SPCT. Whilst challenging, planning end-of-life care outside of hospital is deliverable, clinically appropriate in a subset of patients with concomitant SARS-CoV-2 infection and cancer, and supports patient and family preferences for care delivery.

It is important to acknowledge a number of limitations to our study. OnCovid is a retrospective study and appraisal of the sources of patient data shows a clear imbalance of SPCT data, where four centres (one in Spain and three in the UK) contributed to >75% of the patients. SPCT referral data in this study were in fact mainly collected from tertiary cancer centres in London, UK and Barcelona, Spain. Not all the centres involved in the OnCovid study group had the capacity to input SPCT data. This could limit the generalisation of our findings and reflect improved access to SPCT services in these cities. The majority of patients enrolled in this sub-study were from the UK, which is known for its high standards in end-of-life care, with comprehensive national policies and a strong hospice movement.^44^ Therefore, the practices described in this investigation may disproportionately reflect practice within the UK than in other European countries. In addition, the provision of symptomatic care and SPCT capacity may be different across these countries. Furthermore, the data presented focus on patients managed within large tertiary hospitals, and there may be valuable lessons to be learnt from the challenges faced in SPCT provision in smaller centres and in the community setting.^45^

The aim of this study was not to prospectively assess patient characteristics leading to referral to SPCT and subsequently compared outcomes. This retrospective analysis is a description of referral patterns to SPCT. The key part of our analysis was to attempt and describe reasons for referral and symptomatic needs of patients so that clinical services can subsequently capitalise on these data in the context of an unresolved pandemic. Sufficiently powered prospective studies may help understand any statistical significance differences between the outcomes for patients referred to SPCT and those who were not. Furthermore, prospective studies may facilitate better understanding of the decision-making processes clinicians use when referring patients to SPCTs.

In conclusion, this study describes the challenges of implementing SPCT in patients with COVID-19 and cancer and highlights the value of SPCT involvement in the management of these patients. We found that patients accessing SPCT support often have a higher number of co-morbidities, higher tumour burden and complex clinical needs. We have shown that the multifaceted role of SPCTs extends beyond symptom control as it frequently embraces broader roles, including assistance with complex clinical decision making, discharge planning, end-of-life care and psychological support. We found SPCT referral for psychological concerns to be at a higher rate than elsewhere reported, raising important questions about the availability of adequate psychosocial support for patients and their families. End-of-life was characterised by high symptomatic burden, suggesting the need for specialist oversight of pharmacological and non-pharmacological interventions to best support deteriorating patients. Therefore, integration of SPCTs in the management of patients with cancer and COVID-19 is necessary to provide equitable, specialist care for this vulnerable population.

## Supplemental Material

sj-docx-1-tam-10.1177_17588359211042224 – Supplemental material for Specialist palliative and end-of-life care for patients with cancer and SARS-CoV-2 infection: a European perspective Click here for additional data file. Supplemental material, sj-docx-1-tam-10.1177_17588359211042224 for Specialist palliative and end-of-life care for patients with cancer and SARS-CoV-2 infection: a European perspective by Gehan Soosaipillai, Anjui Wu, Gino M Dettorre, Nikolaos Diamantis, John Chester, Charlotte Moss, Juan Aguilar-Company, Mark Bower, Christopher CT Sng, Ramon Salazar, Joan Brunet, Eleanor Jones, Ricard Mesia, Amanda Jackson, Uma Mukherjee, Ailsa Sita-Lumsden, Elia Seguí, Diego Ottaviani, Anna Carbó, Sarah Benafif, Rachel Würstlein, Carme Carmona, Neha Chopra, Claudia Andrea Cruz, Judith Swallow, Nadia Saoudi, Eudald Felip, Myria Galazi, Isabel Garcia-Fructuoso, Alvin J. X. Lee, Thomas Newsom-Davis, Yien Ning Sophia Wong, Anna Sureda, Clara Maluquer, Isabel Ruiz-Camps, Alba Cabirta, Aleix Prat, Angela Loizidou, Alessandra Gennari, Daniela Ferrante, Josep Tabernero, Beth Russell, Mieke Van Hemelrijck, Saoirse Dolly, Nicholas J Hulbert-Williams, David J Pinato in Therapeutic Advances in Medical Oncology
