# Supplementary material for: Specialist palliative and end-of-life care for patients with cancer and SARS-CoV-2 infection: a European perspective
Source: Ther Adv Med Oncol. 2021 Sep 2;13:17588359211042224. doi: 10.1177/17588359211042224 (PMC8419540; doi:10.1177/17588359211042224)
Supplement: sj-docx-1-tam-10.1177_17588359211042224 – Supplemental material for Specialist palliative and end-of-life care for patients with cancer and SARS-CoV-2 infection: a European perspective [file sj-docx-1-tam-10.1177_17588359211042224.docx]

**Supplementary Table 1: Distribution of SPCT+ and SPCT- groups.**

| **Study site** | **SPCT+**  ***n*=206** | **SPCT- *n*=349** |
| --- | --- | --- |
| **United Kingdom**  Barts Health NHS Trust (London)  Chelsea and Westminster Hospital NHS Foundation Trust (London)  Guy’s and St Thomas’ NHS Foundation Trust (London)  Imperial College Healthcare NHS Trust (London)  University College London Hospitals NHS Foundation Trust (London)  Velindre Cancer Center (Cardiff) | **122**  29  3  40  9  39  2 | **277**  95  15  89  10  55  13 |
| **Spain**  Catalan Institue of Oncology (Girona)  Hospital Clínic de Barcelona (Barcelona)  ICO L’Hospitalet de Llobregat (Barcelona)  Institut Català d’Oncologia Badalona (Barcelona)  Vall d’Hebron University Hospital (Barcelona) | **77**  4  1  60  11  1 | **56**  7  0  26  15  8 |
| **Belgium**  Institut Jules Bordet (Brussels) | **6**  6 | **13**  13 |
| **Germany**  Medical Center of the University of Munich (Munich) | **1**  1 | **3**  3 |

SPCT: Specialist palliative care team; NHS: National Health Service; ICO: Catalan Institute of Oncology

**Supplementary Table 2: Ethics board approval by site.**

| **Study site** | **Ethics review board** |
| --- | --- |
| **United Kingdom**  Barts Health NHS Trust (London)  Chelsea and Westminster Hospital NHS Foundation Trust (London)  Guy’s and St Thomas’ NHS Foundation Trust (London)  Imperial College Healthcare NHS Trust (London)  University College London Hospitals NHS Foundation Trust (London)  Velindre Cancer Center (Cardiff) | Central ethical approval by the Health Research Authority (20/HRA/1608) |
| Catalan Institute of Oncology (Girona) | Catalan Institute of Oncology Institutional Review Board |
| Hospital Clínic de Barcelona (Barcelona) | Clinical Research Ethics Committee of the Hospital Clínic de Barcelona |
| ICO L’Hospitalet de Llobregat (Barcelona) | Catalan Institute of Oncology Institutional Review Board |
| Institut Català d’Oncologia Badalona (Barcelona) | Catalan Institute of Oncology Institutional Review Board |
| Vall d’Hebron University Hospital (Barcelona) | The Hospital Universitario Vall d’Hebron Clinical Research Ethics Committee |
| Institut Jules Bordet (Belgium) | Jules Bordet Institute Ethics Committee |
| Medical Center of the University of Munich (Munich) | The Ethics Committee of the University Hospital of Munich |

NHS: National Health Service; HRA: Health Research Authority; ICO: Catalan Institute of Oncology
